# A New Player in the Game: Can Exergame Be of Support in the Management of Atrial Fibrillation?

**DOI:** 10.3390/medicina60010172

**Published:** 2024-01-17

**Authors:** Donato Giuseppe Leo, Riccardo Proietti

**Affiliations:** 1 Department of Cardiovascular and Metabolic Medicine, Institute of Life Course and Medical Sciences, Faculty of Health and Life Sciences, University of Liverpool, Liverpool L7 8TX, UK; 2 Liverpool Centre for Cardiovascular Sciences, Liverpool Heart and Chest Hospital, University of Liverpool, Liverpool L8 7TX, UK

**Keywords:** atrial fibrillation, e-health, exercise, exergame, physical activity

## Abstract

Atrial fibrillation (AF) is the most common form of cardiac arrhythmia, currently affecting 2–3% of the world’s population. Traditional exercise and physical activity interventions have been successfully implemented in the management of AF, with the aim of improving patients’ quality of life and their exercise capacity, as well as reducing their mortality rate. Currently, new technology-mediated approaches to exercise, defined as exergame, have been shown to be successful in the delivery of exercise home-based interventions in patients with cardiovascular diseases. However, data on the effects of exergame on AF are not yet available. In this paper, we summarise the current literature on the role of traditional exercise in AF and how it affects the pathophysiology of this condition. We also review the current literature on exergame and its employment in cardiac rehabilitation and suggest its potential role in the management of AF patients. A review of the evidence suggests that traditional exercise (of light-to-moderate intensity) is beneficial in patients with AF. Additionally, exergame seems to be a promising approach for delivering exercise interventions in patients with cardiovascular diseases. Exergame may be a promising tool to improve the quality of life and exercise capacity in patients with AF, with the additional advantage of being remotely delivered, and the potential to increase patients’ engagement. Proper guidelines are required to prescribe exergame interventions, considering the principles of traditional exercise prescription and applying them to this new e-health approach. Further studies are needed to validate the use of exergame in patients with AF.

## 1. Introduction

With 2–3% of the world’s population affected, atrial fibrillation (AF) is the most common arrhythmia, inducing the rapid and irregular beating of the heart’s atrial chambers [1]. Known risk factors for the onset of AF are hypertension, coronary artery disease, congenital heart disease, obesity, diabetes mellitus, thyrotoxicosis, sleep apnea, chronic obstructive pulmonary disease (COPD), smoking, and excessive alcohol consumption [2,3,4,5]. The management of AF has embraced a holistic and integrated care approach (ABC—Atrial Fibrillation Better Care—pathway), following a step-wise assessment of the patient [6,7].

Exercise and physical activity play an important role in the prevention of cardiovascular disease [8], and they have been proven to be effective in improving exercise capacity and quality of life in patients with AF, thereby reducing the mortality rate in this cohort [9,10]. Furthermore, exercise-based cardiac rehabilitation has been shown to be associated with a better prognosis in patients with AF [11].

In recent years, with the advent of more sophisticated game technology, a new entertainment style of exercise and physical activity has emerged, called exergame or active video game. Exergame is defined as any form of video game software that requires physical exertion in order to be played [12]. This approach to exercise is increasing in popularity not only in the entertainment market but also in the field of exercise-based rehabilitation [13], proving to be potentially effective in increasing the physical activity level of older adults [14] and their cardiovascular fitness [13,15]. However, some limitations on the employment of exergame in the healthcare contest are still present. Although some beneficial effects of exergame on cardiovascular fitness and on the overall quality of life of patients with cardiovascular diseases have been proven, this modality has yet to be taken into consideration as an exercise-based rehabilitation model for patients with AF. Therefore, the aim of this review is to highlight the role that exergame plays in exercise-based interventions for cardiovascular diseases, its limitations, and the potential applications of this approach in improving cardiovascular outcomes and quality of life in patients with AF.

## 2. Select Player One: Exercise-Based Interventions in the Management of AF

**Figure 1:**
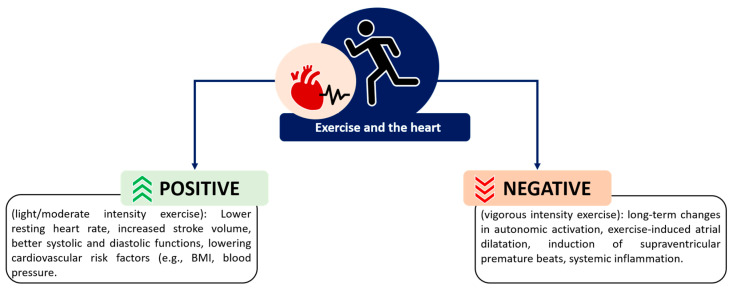
Positive and negative effects of exercise at different intensities on the heart.

Regular exercise induces beneficial cardiac adaptations (e.g., lower resting heart rate, increased stroke volume, and better systolic and diastolic functions) [16,17], and also positively affects BMI, glucose and lipid control, and blood pressure, which are all known risk factors for AF [17,18] (Figure 1). Patients’ activity levels can be negatively affected by the burden of AF [19]. Nevertheless, exercise-based cardiac interventions have been proven to be effective in increasing the quality of life and the exercise capacity of patients with AF (Table 1). A randomised-controlled trial [20] conducted in Canada on a sample of 81 patients with nonvalvular AF showed that a 6-month exercise and nutritional intervention consisting of a home-based physical activity plan of 200 min/week (month 1 to 6), including 2 weekly sessions of supervised cardiac rehabilitation (month 4 to 6), improved the quality of life in this cohort. Similarly, a randomised controlled study [21] conducted in Denmark on a sample of 52 patients with paroxysmal or persistent AF showed that a 6-month exercise intervention consisting of two weekly sessions of supervised cardiac rehabilitation (with at least 30 min of aerobic exercise at ≥70% of maximum exercise capacity) improved the quality of life of these patients compared to standard care. The same study also showed an improvement in the exercise capacity in the intervention group [21]. Another randomised controlled study [22] conducted in Denmark on a sample of 47 patients with permanent AF showed that a 12-week exercise intervention consisting of 1 h three times per week of supervised training improved patients’ overall quality of life compared to the baseline. The study also showed that the intervention increased the exercise capacity and reduced the resting pulse rate of the participants [22].

Furthermore, a randomised controlled study [23] conducted in Norway on a sample of 28 patients with chronic AF showed the positive effects of a 2-month exercise program (consisting of 24 training sessions—1.25 h × 3 days/week—of aerobic exercise and muscular strengthening) on the quality of life of these patients compared to usual care. The study also showed that exercise capacity increased by 41% (±36%) in the intervention group [23].

The mortality rate was also reduced in patients with AF when they participated in exercise-based interventions. Indeed, a retrospective cohort study [24] conducted on an international dataset of 1,366,422 patients with AF showed that exercise-based cardiac rehabilitation is associated with 68% lower odds of all-cause mortality.

However, it is important to highlight that only light-to-moderate exercise intensity is recommended in patients with AF, with vigorous-intensity exercise exhibiting potential detrimental effects [17,25]. The remodelling of the heart induced by exercise, known as ‘athlete’s heart’, does extend the normal cardiac dimension and functions (remodelling of the heart), causing difficulties in discriminating between changes due to the cardiac adaptations to exercise and pathophysiological changes (such as arrhythmogenic cardiomyopathy) [26]. This is especially true when taking into account hypertrophic cardiomyopathy (a myocardial disease caused by gene mutations that induce a hypertrophied left ventricle), of which electrocardiogram (ECG) and echocardiographic findings often overlap with the athlete’s heart [27]. Changes that fall under the athlete’s heart include bradycardia, cardiac hypertrophy, and ECG abnormalities [28]. The modality of exercise strongly influences the risk of developing AF, with athletes of mixed sports being at a higher risk of developing AF compared to athletes of endurance sports [29]. Interestingly, it seems that the risk of developing AF is greater in younger athletes (<55 years old) compared to older athletes [29], but the mechanisms behind this are not yet clear. Men regularly engaging in vigorous-intensity exercise have been shown to have a 12% increased risk of developing AF [30]. In women, on the contrary, vigorous exercise seems to have a protective effect against the onset of AF [30]. The relationship between high-intensity exercise and the risk of AF is still not fully understood [17]. However, potential causes may be related to vigorous exercise inducing more long-term changes in autonomic activation [17,31], exercise-induced atrial dilatation [17,31,32], induction of more supraventricular premature beats [17,33], and systemic inflammation [17,34] (Figure 1). Suggested explanations for gender differences relate to men being potentially more affected by vigorous exercise due to their large atria and for having more exercise-induced remodelling of the heart [35].

In addition, when considering exercise interventions for AF patients, it is also important to consider secondary forms of AF, as different pathophysiological mechanisms, the presence of comorbidities, and polypharmacy are all factors that need to be taken into account in the prescription of exercise-based rehabilitation [36]. Indeed, patients with AF are often burdened by several comorbidities [37], with several studies showing an association between various conditions (such as hyperthyroidism [38] or channelopathies [39,40]) and an increased risk of developing AF. Emotional stress and anxiety also play important roles in the onset of AF [41,42] due to their negative impact on the hypothalamic–pituitary–adrenocortical axis [43,44]. Furthermore, lifestyle changes (e.g., alcohol intake and smoking cessation) are highly relevant for the prevention and management of AF [45], with increased physical activity and reduction in sedentary behaviour only partially accountable for the improved management of these patients, for whom a more holistic approach is highly beneficial [46,47].

## 3. Levelling-Up: Exergame as an Intervention for Cardiovascular Diseases

Few studies [48,49,50,51,52] have assessed the efficacy of exergame as a tool to improve traditional exercise-based cardiac rehabilitation (Table 2). The advantage of exergame over most traditional cardiac rehabilitation programs is the possibility for the patients to conduct the intervention at home [53] using a normal video game hardware (e.g., game console), reducing National Health System (NHS) costs [54], and reducing barriers to access rehabilitation for patients (e.g., travel distance and schedule flexibility) [55]. The risks of adverse events associated with home-based cardiac rehabilitation have been deemed to be low [56].

A pre–post test study [48] conducted in Jamaica on a sample of 28 patients with different cardiovascular diseases (mainly hypertension—68% and coronary artery disease—79%) showed that a 6-week exergame intervention consisting of 3 × 40 min/week training sessions with the Nintendo Wii Fit Plus software can improve functional endurance in this cohort. Another randomised controlled study [50] conducted in Spain on 20 patients with ischemic heart disease showed that 8 weeks of exergame consisting of 2 × 60 min/week aerobic sessions using the Microsoft XBOX with the Kinect sensor improved exercise capacity and quality of life and reduced depression in the intervention group. However, the results did not show statistically significant differences compared to the traditional exercise intervention (control group) (*p >* 0.05) [50]. A pilot study [49] conducted in Sweden on a sample of 32 patients with heart failure showed that an exergame intervention of 12 weeks consisting of 20 min × day of playing the Nintendo Wii Sports software was successful in increasing the exercise capacity of 53% of this cohort. However, an international randomised controlled trial [52] conducted on a sample of 605 patients with heart failure engaged in a 12-month exergame intervention consisting of 5 × 30 min weekly sessions with the Nintendo Wii Sports software showed no statistically significant differences in exercise capacity compared with traditional physical activity (control group). Also, another randomised controlled study [51] conducted in Portugal on a sample of 33 patients who completed phase II cardiac rehabilitation and underwent a 6-month home-based exergame intervention consisting of 3 × 60–90 min weekly sessions using the Microsoft XBOX Kinect did not find any statistically significant differences in terms of improved quality of life and reduced depression compared with traditional exercise (group 2—performing the same exercise protocol without the Kinect) or usual care (group 3—control group) (*p >* 0.05).

The differences in results between the above-mentioned studies can be explained by different factors, which also extend to the literature concerning exergame interventions for other health conditions. Firstly, it is important to note that there is confusion in the literature about the term “exergame” [57]. Indeed, exergame is not always associated with a specific exercise intervention, but rather with a variety of interventions that target different components of physical fitness (e.g., strength, flexibility, and endurance) [57]. There are studies that define, using the term “exergame”, interventions that use only virtual reality as a supplement to traditional exercise/physical activity [15], whereas other studies have used the term to define mobile applications that simply promote physical activity (e.g., increasing the daily step count) [58,59]. In our synthesis, we have only included studies that use exergame interventions based on hardware/software that requires physical exertion to be played (e.g., using motion control to play a simulated tennis game, or using a camera to recognise body movement and translate it to in-game actions) [48,49,50,51,52].

Studies that have assessed the efficacy of exergame on health-related outcomes show a high heterogeneity between the devices used to deliver the intervention, which also affects the type of exercise administered: game console, mobile phone, virtual reality visor, and dedicated hardware platform [60,61,62,63,64]. Additionally, the intensity and frequency of the exergame intervention are not always taken into account, as acknowledged by Jaarsma et al. (2021) [52] in the discussion of the findings of their study. This is a major limitation of studies assessing the efficacy of exergame in improving cardiovascular outcomes, as frequency and intensity are key components of an exercise program and are highly relevant to cardiac rehabilitation [65,66]. Moreover, differences in the study design, sample size, and duration of the intervention can all play a significant role in the final results.

## 4. Let’s Start the Game: Potential Role of Exergame in the Management of AF

Exercise and physical activity can improve the quality of life and exercise capacity in patients with AF [20,21,22,23] and also reduce their mortality rates [24]. Exergame has shown promise in delivering similar effects to traditional exercise, improving the quality of life and exercise capacity in patients with cardiovascular disorders [48,49,50]. Moreover, exergame has also the potential for more entertaining interventions, which may increase treatment adherence and reduce potential barriers to accessing cardiac rehabilitation [55,67]. However, so far exergame interventions have often neglected the importance of frequency, intensity, time (duration), and type of exercise that are essential for the success of an exercise program [68]. Moreover, the heterogeneity of the platforms and software used to deliver the intervention may lead to poor outcomes. Clear guidelines for exergame prescription should be identified before attempting to evaluate the effectiveness of this intervention in cardiac rehabilitation and, more specifically, in the management of patients with AF. Exergame can be a promising tool to enhance exercise-based cardiac rehabilitation, with the potential to be administered both in clinical settings and in-home settings. With the rapid advancement of technology, the integration of exergame platforms with wearable health sensors [69] and the Internet of Things (IoT) [70] may lead to better clinical management, thereby allowing the clinical team to remotely monitor the progress of the training program, making rapid adjustments where necessary. Exergames can also be played together with family members in the comfort of the patient’s home, potentially increasing adherence and adding enjoyment to the treatment [67]. The innovation of exergame interventions in the management of AF may be game changing, especially when considering the importance of home-based rehabilitation interventions in light of the recent COVID-19 pandemic and the consequent lockdown [71]. However, a proper definition of the essential parameters of an exergame intervention is fundamental before attempting further studies to assess its effectiveness.

As a final note, some limitations in relation to our synthesis are noteworthy. First, as a narrative review, it lacks a systematic approach, and some relevant studies may have been potentially overlooked and excluded. It is also important to note that the quality of the included studies varied markedly in terms of methods, design, and sample size, and that no formal quality assessment of these studies was completed.

## 5. Conclusions

The increasing incidence of AF is a worldwide burden. Exercise of light to moderate intensity has been shown to play a positive role in the management of AF, thereby improving the quality of life of patients and their exercise capacity and reducing the mortality rate in this cohort. Exergame is a new approach to exercise and physical activity interventions, which has already been implemented in exercise-based cardiac rehabilitation for patients with cardiovascular diseases, showing promising potential. Despite the increasing popularity of exergame in healthcare and cardiovascular rehabilitation, no studies have investigated the effects of this approach in patients with AF. The advantage of exergame compared to traditional exercise interventions is the possibility of planning personalised, home-based interventions that may also facilitate patients’ adherence to treatment. However, clear guidelines on exergame prescription, especially focusing on frequency, intensity, time (duration), and type of activity, should be properly defined before attempting studies aiming to show exergame effectiveness in AF patients.

## Figures and Tables

**Table 1:** Summary of characteristics of the included studies related to exercise interventions for atrial fibrillation diseases (*n* = 5).

Study ID, Year, Country	Design, Study Population	Intervention	Outcome(s)	Results	Conclusion
**[20] Bittman**, 2022, Canada	RCT; Non-permanent, nonvalvular AF; Intervention *n* = 34 (mean age 63.7 ± 8.6 years, female *n* = 11); Control *n* = 38 (mean age 61.0 ± 9.7 years, female *n* = 17).	Nutritional plan and exercise program with 200 min/week (month 1 to 6) plus 2 weekly sessions of supervised cardiac rehabilitation (month 4 to 6)	QoL (SF-36)	**QoL:** [Intervention (I), Control (C), mean ± SD—vitality I: 13.2 ± 20.4; C: 1.0 ± 14.9, *p* < 0.001; social functioning I: 14.7 ± 24.1; C: 2.4 ± 21.2, *p* = 0.018; emotional well-being I: 5.5 ± 14.1; C: −1.0 ± 13.3, *p* = 0.017; and general health perceptions I: 8.1 ± 12.3; C: 2.7 ± 13.3, *p* = 0.009].	(+) exercise training improved QoL in AF patients.
**[23] Hegbom**, 2007, Norway	RCT; Chronic AF; Intervention *n* = 13 (mean age 62 ± 7, female *n* = 13); Control *n* = 15 (mean age 65 ± 7, female *n* = 13).	24 training sessions (1.25 h × 3 days/week) of aerobic exercise and muscular strengthening	QoL (SF-36); exercise capacity (Borg Scale 17)	**QoL**: [mean score ± SD, physical functioning pre 82 ± 14 vs. 86 ± 10 post, *p* = 0.01; bodily pain pre 82 ± 17 vs. post 92 ± 14, *p* = 0.01; vitality pre 61 ± 14 vs. 68 ± 13 post, *p* = 0.01; and role-emotional pre 85 ± 28 vs. post 94 ± 20, *p* = 0.01]. **Exercise capacity**: increased by 41% (±36%)	(+) exercise training improved QoL and exercise capacity in AF patients.
**[21] Joensen**, 2019, Denmark	RCT; Paroxysmal or persistent AF; Intervention *n* = 28 (mean age 62.2 ± 10.0, female *n* = 11); Control *n* = 24 (mean age 60.2 ± 8.9, female *n* = 7).	6-month exercise intervention consisting of two weekly sessions of supervised cardiac rehabilitation (with at least 30 min of aerobic exercise at ≥70% of maximum exercise capacity).	QoL (AF-QoL-18; exercise capacity (ergometer cycle test)	**QoL**: [Intervention (I), Comparator (C), mean ± SD—I: baseline 48.4 ± 22.8 to 6 months 68.0 ± 15.2, vs. C: baseline 51.6 ± 22.3 to 6 months 59.2 ± 27.3, *p* = 0.031]. **Exercise capacity**: [Intervention: mean ± SD, 176 ± 48 pre vs. 190 ± 55 at 6 months, *p* = 0.026]	(+) exercise training improved QoL and exercise capacity in AF patients.
**[22] Osbak**, 2012, Denmark	RCT; Permanent AF; Intervention *n* = 24 (mean age 69.5 7.3, female *n* = 6); Control *n* = 23 (mean age 70.9± 8.3, female *n* = 6).	12-week exercise intervention (1 h/3 times per week of supervised training)	QoL (SF-36; MLHF-Q); exercise capacity (6MWT)	**QoL**: [mean score ± SD, SF-36: physical functioning pre 82 ± 14 vs. 86 ± 10 post, *p* = 0.01; bodily pain pre 82 ± 17 vs. post 92 ± 14, *p* = 0.01; vitality pre 61 ± 14 vs. 68 ± 13 post, *p* = 0.01; role-emotional pre 85 ± 28 vs. post 94 ± 20, *p* = 0.01; and MLHF-Q: *p* = 0.03]. **Exercise capacity**: [mean score(meters), SD, Intervention (504.4 ± 85.1 pre vs. 569.9 ± 92.6 post) vs. control (453.1 ± 100.1 pre vs. 454.1 ± 95.7 post), *p* = 0.001]. intervention decreased patients’ **resting pulse** [mean ± SD: 94.8 ± 22.4 to 86.3 ± 22.5 beats/min, *p* = 0.049].	(+) exercise training improved QoL and exercise capacity and decreased resting heart rate in AF patients.
**[24] Buckley**, 2021, UK	Retrospective study on international database	N/A	Mortality	**Mortality**: 68% lower odds of all-cause mortality [odds ratio: 0.32; 95% CI: 0.29–0.35].	(+) exercise training reduced the mortality rate in AF patients.

6 MWT = 6 min walking test; AF = atrial fibrillation; AF-QoL-18 = health-related quality of life in patients with atrial fibrillation; CI = confidence interval; N/A = not applicable; QoL = quality of life; RCT = randomised controlled trial; SD = standard deviation; SF-36 = Short Form 36-item; MLHF-Q = Minnesota Living with Heart Failure Questionnaire. + indicates the positive effect of the intervention.

**Table 2:** Summary of characteristics of the included studies related to exergame interventions for cardiovascular diseases (*n* = 5).

Study ID, Year, Country	Design, Study Population	Intervention	Outcome(s)	Results	Conclusion
**[50] Garcia-Bravo**, 2020, Spain	RCT; Ischemic heart disease; Intervention *n* = 10 (mean age 48.7 ± 6.66, gender not reported); Control *n* = 10 (mean age 53.7 ± 10.3, gender not reported).	8 weeks of exergame consisting of 2 × 60 min/week aerobic sessions using the Microsoft XBOX with the Kinect sensor	Exercise capacity (6MWT); QoL (SF-36); depression level (Beck-II depression inventory)	**Exercise capacity**: [mean ± SD, distance: 457.80 ± 132.00 pre vs. 513.00 ± 117.00 post, *p* = 0.005. **Quality of life and level of depression**: SF-36 general health: *p* = 0.049, SF-36 social function: *p* = 0.010, Beck-II depression inventory: *p* = 0.012].	(+) exergame improved exercise capacity and quality of life and reduced the level of depression.
**[52] Jaarsma**, 2021, Sweden, Italy, Israel, the Netherlands, Germany and the USA	International Multicentre RCT; Heart failure; Intervention *n* = 305 (mean age 66 ± 12, female *n* = 85); Control *n* = 300 (mean age 67 ± 11, female *n* = 90).	12-month, 5 × 30 min weekly sessions with the Nintendo Wii Sports software	Exercise capacity (6MWT); self-reported PA level; patients outcome measures	No statistically significant differences between groups [*p* > 0.05].	(=) exergame did not show statistically significant effects compared to traditional exercise.
**[49] Klompstra**, 2014, Sweden	Pilot study; Heart Failure; *n* = 32 (mean age 63 ± 14, female *n* = 10);	12-week, 20 min × day session using the Nintendo Wii Sports	Exercise capacity (6MWT)	**Exercise capacity**: [mean ± SD: 501 ± 95 m pre vs. 521 ± 101 m post, *p* < 0.05].	(+) exergame improved exercise capacity.
**[48] Nelson**, 2014, Jamaica	Single group pre-post test; Cardiac disease; *n* = 28 (mean age 62.1 ± 11.4, female *n* = 15).	6 weeks consisting of 3 × 40 min/week training sessions with the Nintendo Wii Fit Plus software	Exercise capacity (6MWT)	**Exercise capacity**: [mean ± SD, from 461.93 m (SD 5 105.87) pre to 498.22 m (SD 5 132.95) post, *p* < 0.001].	(+) exergame improved exercise capacity.
**[51] Vieira**, 2018, Portugal	RCT; Patients who completed phase II cardiac rehab; Exergame *n* = 11 (mean age 55 9.0, gender not reported); Booklet group *n* = 11 (mean age 59 11.3, gender not reported); Control group *n* = 11 (mean age 59 5.8, gender not reported).	6-month, 3 × 60–90 min weekly session using the Microsoft XBOX Kinect	QoL (MacNew questionnaire); depression, anxiety, and stress (Depression, Anxiety, and Stress Scale 21)	No statistically significant differences between groups [*p* > 0.05]	(=) exergame did not show statistically significant effects compared to control (traditional exercise; usual care).

6 MWT = 6 min walking test; PA = physical activity; QoL = quality of life; RCT = randomised controlled trial; SD = standard deviation; SF-36 = short form 36-item. + indicate positive effect of the intervention, and no difference (=) between the intervention and the control group.

## Data Availability

Not applicable.
